# Novel 3D printed resin crown versus prefabricated zirconia crown for restoring pulpotomized primary molars: in vitro evaluation of fracture resistance and marginal gap

**DOI:** 10.1007/s40368-025-01038-1

**Published:** 2025-05-02

**Authors:** E. I. A. Elnagar, G. G. Allam, N. M. A. Khattab

**Affiliations:** Department of Pediatric Dentistry, Faculty of Dentistry, Ain Shams University, Organization of African Unity Street, Cairo, Egypt

**Keywords:** 3D dental printer, Primary molars, Prefabricated zirconia crown, Fracture resistance

## Abstract

**Aim:**

This in vitro study aimed to evaluate the marginal gap and fracture resistance of 3D printing microfilled hybrid resin crowns in comparison to prefabricated zirconia crowns on pulpotomized primary teeth.

**Materials and methods:**

Twenty primary molars were selected for the study and randomly divided into two groups (*n = *10). Group1 received 3D printed microfilled hybrid resin crowns; Group 2 received prefabricated zirconia crowns. To simulate 6 months of oral conditions, thermodynamic cycling was performed, and the marginal gap was measured using a stereomicroscope with digital camera at 40 × magnification. For each sample, eight points along the margins for each axial surface were captured. The fracture resistance of each group was assessed by applying increasing load till crown fracture using a computer-controlled universal testing machine. Data were tested for normality using the Shapiro–Wilk test. Data were analyzed using an independent t test. A significant level was set at *P < *0.05.

**Results:**

Regarding fracture resistance, 3D printed crowns group had statistically significant higher mean values than the prefabricated zirconia crowns group; the values were 1235.97 ± 412.12 N and 576.56 ± 221.53 N, respectively (*P < *0.001). However, there was no significant difference in the marginal gap between the two types of crowns with average 32.00 ± 7.54 for 3D printed crowns and average 34.14 ± 9.79 for zirconia crowns (*P < *0.001).

**Conclusion:**

3D printed microfilled hybrid resin crowns could be a suitable esthetic alternative for restoring pulp-treated primary molars. It is possible to provide an additional esthetic solution for the parents/children to satisfy the need for esthetic restoration of primary molars.

**Clinical trial:**

Not applicable (in vitro study).

## Introduction

Dental caries is highly prevalent in children and adolescents and can lead to dental decay. Restorative dental treatment is critical to maintain the remaining dental structure, restoring chewing function, and preserving the arch length integrity (Lee et al. 2024). American Academy of Pediatric Dentistry recommends full crown coverage in cases of extensive dental caries or following pulp therapy as pulpotomy or pulpectomy (Lee et al. 2024). The most frequently used full-coverage restoration is a preformed metal crown (PMC). PMC is recommended due to its simple preparation, durability, less recurrent caries, and low cost (Agrawal et al. 2022). Despite these benefits, the major drawback is poor esthetics because of its metallic color (Mathew et al. 2020).

Even for children, esthetics is a crucial consideration when restoring severely damaged teeth (Hamrah et al. 2021; Goswami et al.^2024^). While open-faced and pre-veneered preformed metal crowns have been utilized as alternatives to preformed metal crowns, they have numerous drawbacks. Compared with the performance of other crowns, prefabricated zirconia crowns exhibit exceptional strength, biocompatibility, and esthetics compared to other crowns (Ajayakumar et al. 2020). However, there are some clinical limitations and disadvantages for zirconia crowns as they require aggressive tooth reduction and are expensive (Alzanbaqi et al. 2022).

Numerous dental specialties have implemented the use of computer-aided design and computer-aided manufacturing (CAD/CAM). The CAM approach is currently experimented with additive manufacturing, which is also referred to as three-dimensional printing technology (3D printing). The advent of 3D printing technology has significantly enhanced clinical capabilities, enabling practitioners to deliver highly precise, fast, customizable, and esthetically optimized treatments (Jang et al. 2024). Beyond improved accuracy, 3D printing offers advantages such as lower material waste and the ability to create patient-customizable solutions with unparalleled precision. Moreover, 3D printing facilitates cost-effective production of dental crowns, ultimately improving both functional outcomes and patient satisfaction.

One of the most important parameters that must be considered during crown preparation is the marginal fit. Poor marginal fit decreases the durability of the crown, leading to microleakage, secondary caries, and gingival inflammation. Fracture resistance is also one of the key factors, which determine the survival of the crown and its ability to withstand occlusal forces.

Elastic modulus is a key factor for full coverage restoration of the deciduous molars. Composite resin crowns are elastic crowns that are expected to bear the stresses induced by the prepared tooth convexity during the crown insertion (Park et al. 2023).

Due to the large range of dental restorative materials available, choosing the most suitable material for a given case can often be challenging for pediatric dentists. Factors such as fracture resistance and marginal gap, among other factors, can impact the long-term success of dental restoration.

Continuous advancement in 3D printing technology and printable materials enables the fabrication of long-lasting interim and final prostheses that can withstand high stress in the oral cavity (Schweiger et al. 2021).

Therefore, this study aimed to evaluate the marginal gap and fracture resistance of 3D printing resin crowns in comparison to prefabricated zirconia crowns on extracted primary teeth. The null hypothesis is that there are no statistically significant differences between the two types of crowns in terms of fracture resistance and marginal fit.

## Materials and methods

### Sample size estimation

A power analysis was designed to have adequate power to apply a two-sided statistical test of the null hypothesis that there is no difference between the tested groups regarding fracture resistance. By adopting an alpha (α) level of 0.05 (5%), a beta (β) level of 0.2 (i.e., power = 80%), and an effect size (d) of 1.51 calculated based on the results of a previous study (Makawi and Khattab 2019), the predicted total sample size (n) was found to be 16 samples (i.e., 8 samples per group). Sample size calculation was performed using G*Power version 3.1.9.7. (Faul et al. 2007). The sample size was increased by (25%) 20 samples (i.e., 10 samples per group) to account for possible failures in testing.

### Ethics approval

This study was approved by the Research Ethics Committee, Faculty of Dentistry (FDASU-REC). The ethical approval number was FDASU-RECEM122). It was an in vitro study that used teeth collected from anonymous patients.

### Teeth selection

Thirty human carious primary molars were collected from anonymous patients from the Department of Pediatric Dentistry and Dental Public Health, Faculty of Dentistry. The teeth were extracted due to overretention or for orthodontic reasons. Only 20 molars were selected for the study according to the following criteria: free of developmental disorders, no obvious cracks, and no previous dental restorations. For standardization, teeth were selected with an average buccolingual dimension of 6.5 ± 1 mm and mesiodistal dimension of 9.5 ± 1 mm measured using a digital caliper. All soft tissue debris was removed with a hand scaler and then stored in distilled water until usage for a maximum of 1 month (Simsek and Derelioglu 2016).

### Pulpotomy procedure

Caries was removed with a high-speed handpiece using carbide round bur (sizes 4 and 5) under copious irrigation. Then, the access cavity was prepared, and pulp tissue was removed using a large sharp spoon excavator (tip size 1 mm). Then, a thick mix of zinc oxide and eugenol paste was applied to seal the canal orifices, followed by a light cured resin reinforced glass ionomer restorative material (GC Fuji II ®, GC).

### Sample allocation

All samples were allocated randomly by the simple random sampling method using the Research Randomizer software program (https://www.randomizer.org/). An independent person generated randomization codes in sequentially numbered, secured, opaque wrappers to ensure covert distribution into two groups (Mahfouz Omer et al. 2023). Group 1: 3D printed crowns group (*n = *10) was restored by NextDent resin (NextDent C&B MFH, Soesterberg, The Netherlands). Group 2: zirconia crowns (ZC) group (*n = *10)—restored by prefabricated zirconia crowns (NuSmile ZR [NS], NuSmile, Houston, TX, USA).

### Group 1: 3D printed crowns

#### Tooth preparation

The teeth were prepared using high speed contra-angled handpiece with blue-coded tapered diamond stone with round end (Mani TR-12) for buccal, lingual, mesial, and distal walls for 0.7–1 mm, producing a chamfer margin circumferentially, and then reduction of the occlusal surface was done using a blue-coded wheel stone (Mani WR-13) to produce occlusal clearance of 1.5 mm. The samples were then scanned using an intraoral scanner (Cerec Omnicam, Sirona, Bensheim, Germany) (Ahmad et al. 2023) Fig. 1.

**Fig. 1:**
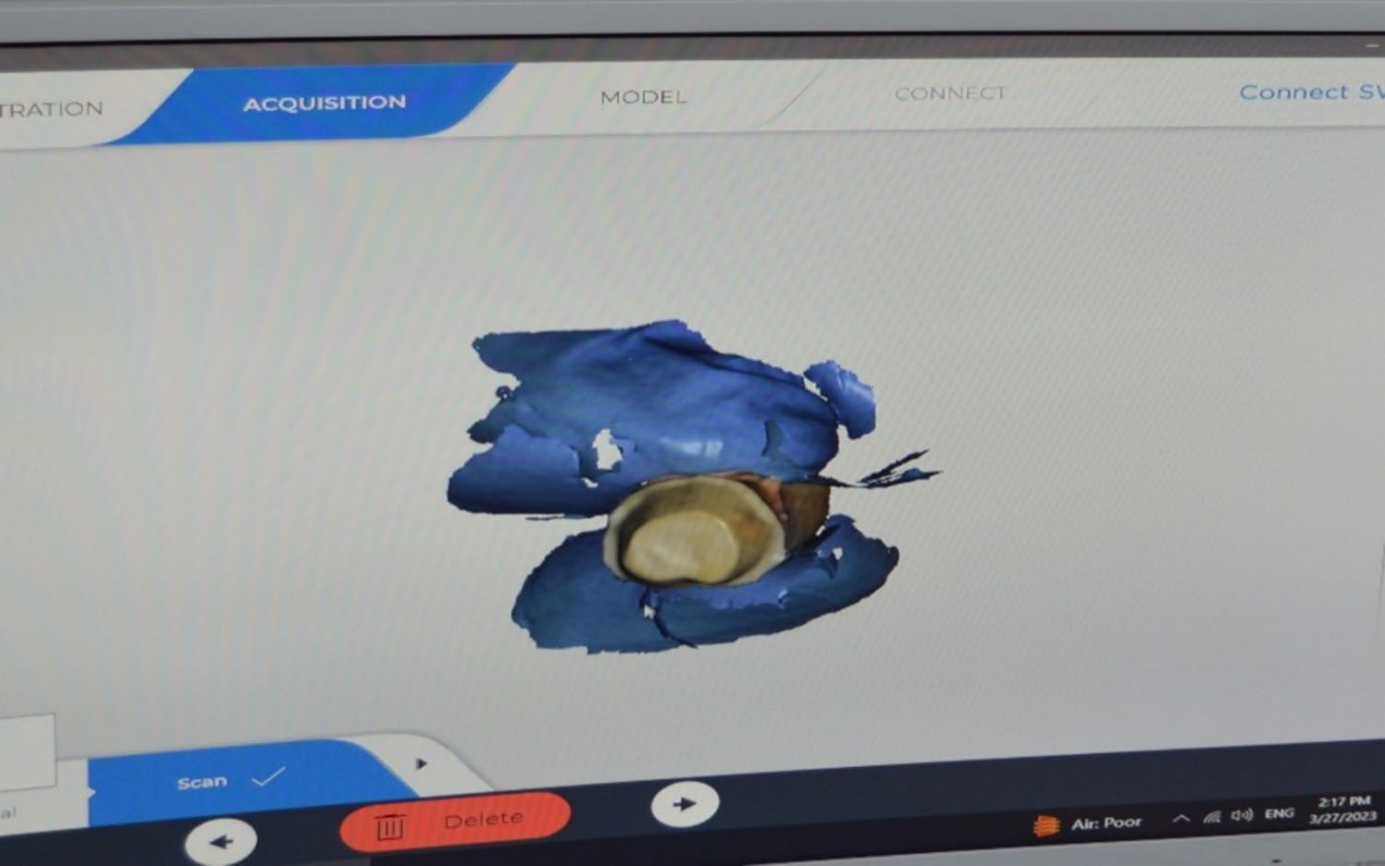
Scanning of a prepared molar using an intraoral scanner

#### 3D crown designing

3D printed resin crowns were designed using the Exocad software (Exocad Gmbh, Darmstadt, Germany) to have a uniform thickness on all surfaces (average 1 mm), including occlusal, buccal, lingual, and proximal surfaces. After reviewing each design, it was exported as a high-resolution STL file to be outsourced and 3D printed (Alshamrani et al. 2023).

MICRODENT 1 PRO printer was utilized to print the 3D printed crowns using NextDent C&B MFH resin via digital light processing (DLP) technology. After printing, the platform was removed from the 3D printer and placed on a paper towel with the printed crowns facing upward. The printed crowns were separated from the platform and rinsed twice in a 96% alcohol solution ultrasonic bath to get rid of any excess material. The duration of each rinse was approximately 3 min to avoid causing defects in the printed crowns. The second rinse was carried out with a clean 96% alcohol solution. After cleaning, the printed crowns were dried to ensure that they were free of alcohol residue (Çakmak et al. 2021).

Printed crowns were then placed in a UV-light curing box with wavelength 300–500 nm (DENTCURE 2 by ZYLO3D) for post-curing according to the manufacturer’s instructions. The printed crowns were finished and polished using a conventional rotary machine.

Each crown was fitted on its prepared molar and assessed according to clinical practice under visual inspection using a 3.5X dental loupes (Lupa Bioart 3.5X) and the dental explorer. The 3D printed crowns were then cemented onto the prepared molars according to the manufacturer’s instructions with resin cement (TOTALCEM).

Excess cement was removed with a sharp dental explorer, and then the samples were stored in distilled water at 37 °C for 24 h (Ahmad et al. 2023).

### Group 2: Zirconia crowns

#### Tooth preparation

The teeth were prepared using high speed contra-angled handpiece with blue-coded tapered diamond stone with a round end (Mani TR-12) for buccal, lingual, mesial, and distal walls for 0.7–1.75 mm, and then reduction of the occlusal surface was performed using a blue-coded wheel stone (Mani WR-13) to produce occlusal clearance of 1.5–2 mm, taking into consideration that the preparation must be free from any undercuts to avoid crown fracture.

The proper size of the crowns was selected using the NUSMILE Zirconia Try-In Kit; then it was removed, and the same size of the crowns as that obtained from the kit was cemented using resin cement (TOTALCEM).

After cementation, excess cement was removed with a hand explorer and the samples were stored in distilled water at 37 °C for 24 h (Ahmad et al. 2023).

### Laboratory steps

#### Thermocycling

The samples were subjected to 5000 thermal cycles with distilled water at 5 °C–55 °C, with dwell time = 25 s and lag time = 10 s using Mechatronic (Germany).

### Marginal gap measurements

**Fig. 2:**
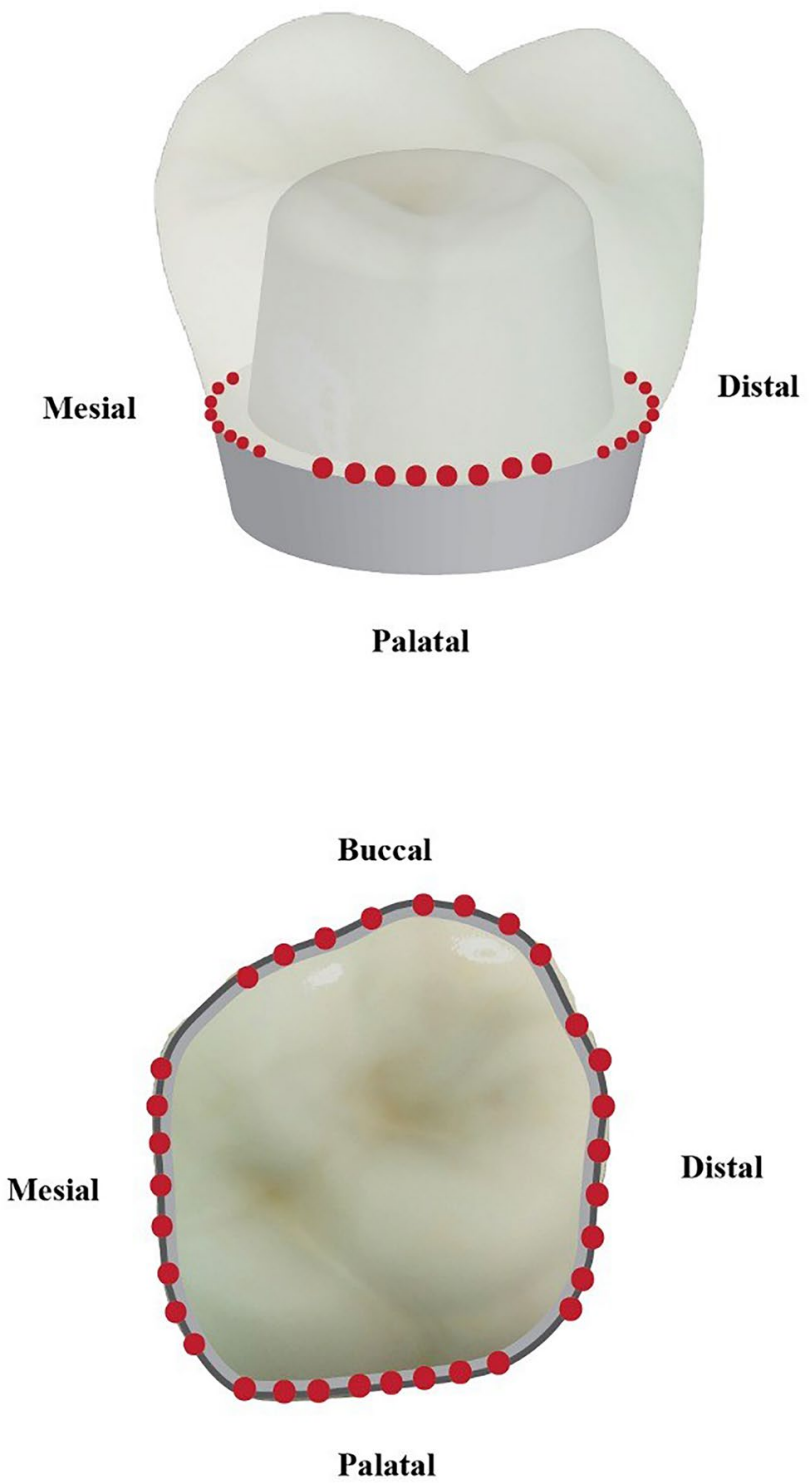
Diagram showing the eight points along the margins for each axial surface of each sample

The samples were examined at the Department of Oral Pathology, Faculty of Dentistry, using a stereomicroscope **(**LG-PS2,Olympus,Japan) with a digital camera (Canon EOS 650D, Japan) at 40× magnification (Al-Haj 2019). The marginal gap was determined using the criteria proposed by Holmes et al., who defined the vertical marginal gap as the distance between the crown margin to the edge of the finish line preparation (Refaie et al. 2023). For each sample, eight points along the margins for each axial surface were captured. Fig. 2. Then, linear measurements were carried out using image analysis software (Image J, 1.41a, NIH, USA). The average marginal gap for each sample was then calculated. The gap distance was measured in micrometers.

### Fracture resistance measurements

#### Preparation of samples for fracture resistance test

Samples were embedded perpendicularly in polyvinyl chloride (PVC) cylinders with the occlusal plane parallel to the ground using self-cure acrylic resin starting 1 mm below the cemento-enamel junction. To simulate the periodontal ligaments, a single layer of Teflon tape was wrapped around the roots of the teeth before being embedded in the acrylic resin.

Each sample was individually mounted on a computer-controlled universal testing machine (Instron Universal Testing Machine, England) and then secured to the lower fixed compartment of the testing machine by tightening screws. An axial loading was applied at the center of the crown using a stainless steel round-ended load applicator (3.6 mm diameter) attached to the upper part of the universal testing machine at a crosshead speed of 1 mm/min (Bani-Hani et al. 2025). The load at failure was manifested by an audible crack and confirmed by a sharp drop in the load–deflection curve recorded in the computer software (Bluehill 3 Universal materials testing software). The load required to fracture was recorded in newton (N) (Beattie et al. 2011).

### Statistical analysis

Numerical data are presented as mean and standard deviation values. They were checked for normality by viewing the distribution and the Shapiro–Wilk's test. Data were found to be normally distributed and analyzed using an independent t test. The significance level was set at *P* < 0.05 within all tests. Statistical analysis was performed with R statistical analysis software version 4.3.1 for Windows (Team RC 2023).

## Results

### Marginal gap (µm)

The intergroup comparison and mean and standard deviation values of the marginal gap (µm) are presented in Table 1.

**Table 1:** Intergroup comparison and mean and standard deviation values of the marginal gap (µm)

Position	Marginal gap (µm) (mean ± SD)		*P* value (ns)
	Zirconia crowns	3D printed crowns	
Buccal	34.80 ± 10.61	26.63 ± 6.70	0.054
Distal	34.62 ± 10.99	37.25 ± 6.51	0.523
Lingual	31.07 ± 7.30	30.56 ± 6.19	0.869
Mesial	36.08 ± 10.64	33.57 ± 7.29	0.545
Overall	34.14 ± 9.79	32.00 ± 7.54	0.277

*P* value < 0.05 is statistically significant

**Fig. 3:**
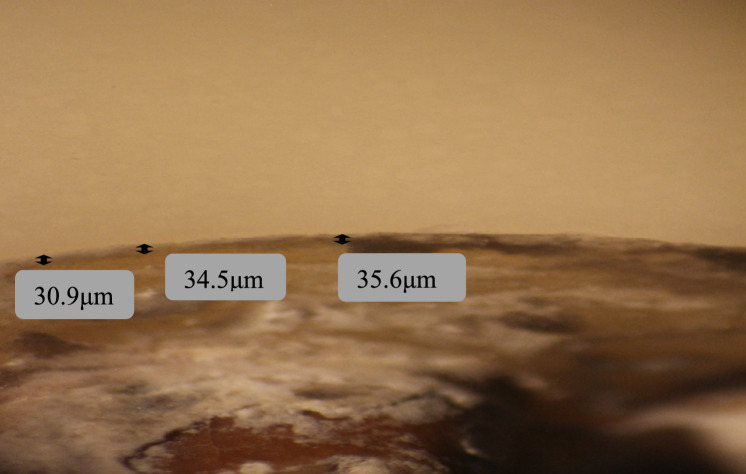
Measurements of the marginal gap using a stereomicroscope for a zirconia crown specimen: the buccal surface

**Fig. 4:**
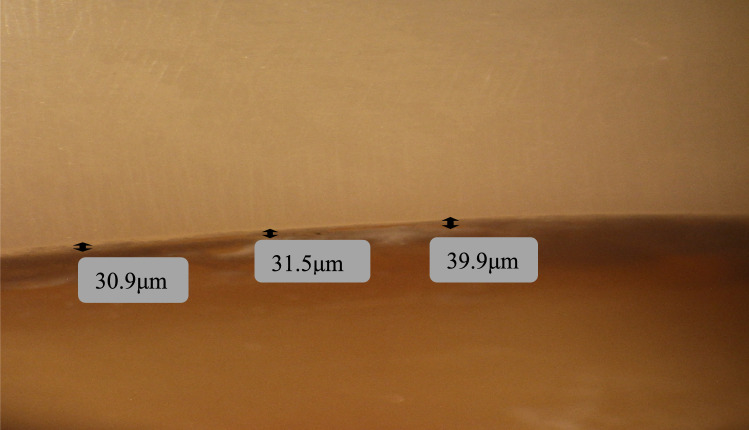
Measurements of the marginal gap using a stereomicroscope for a zirconia crown specimen: the mesial surface

**Fig. 5:**
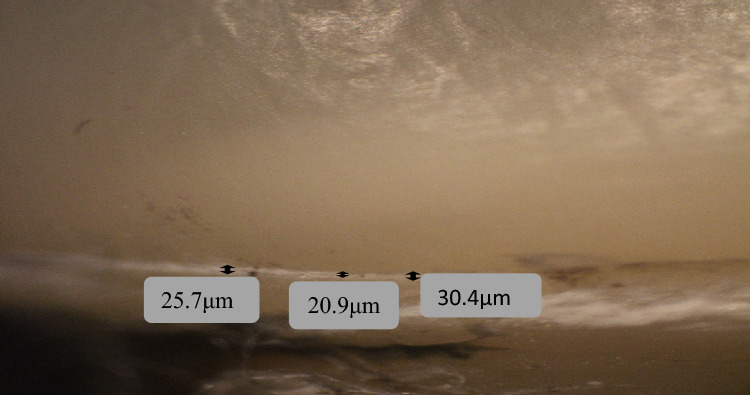
Measurements of the marginal gap using a stereomicroscope for a 3D printed crown specimen: the buccal surface

**Fig. 6:**
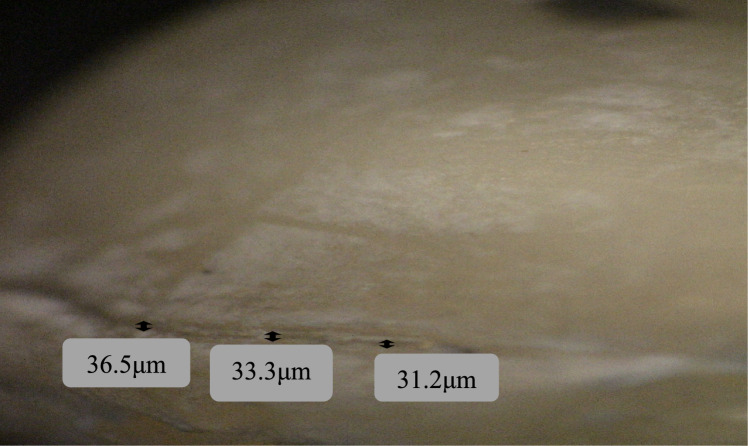
Measurement of the marginal gap using a stereomicroscope for a 3D printed crown specimen: the mesial surface

Zirconia crowns had higher mean values than 3D printed microfilled hybrid resin crowns at the buccal, lingual, and mesial surfaces, while 3D printed crowns had higher values at the distal surface. However, all values were not statistically significant (*P > *0.05) Figs. 3, 4, 5, 6.

### Fracture resistance

The intergroup comparison and mean and standard deviation values of fracture resistance (N) are presented in Table 2.

**Table 2:** Intergroup comparison and mean and standard deviation values of fracture resistance (N)

Fracture resistance (N) (mean ± SD)		*P* value
Zirconia crowns	3D printed crowns	
576.56 ± 221.53	1235.97 ± 412.12	< 0.001*

*P* value < 0.05 is statistically significant

The results revealed that 3D printed microfilled hybrid resin crowns had statistically significant mean values (1235.97 ± 412.12 N) than zirconia crowns (576.56 ± 221.53 N) (*P < *0.001).

## Discussion

Restoring pulpally treated primary molars is a challenge. Although preformed metal crowns are the gold standard treatment option (Kaur et al. 2023), the demand for an esthetic crown for primary molars has increased. Prefabricated zirconia crowns are widely acceptable esthetic restoration option and are the most chosen by pediatric dentists. However, there are multiple variations in the shape and size of the primary teeth, which make it challenging to use ZRCs in the available restricted sizes (Clark et al. 2016; Hanafi et al. 2021).

Restoring primary molars by the use of 3D printing technology may be a novel approach to conventional prefabricated crowns, as 3D printed crowns could be adjusted in terms of shape and size for each patient (Kim et al. 2022). However, research for its use as primary teeth is limited (Al-Halabi et al.^2020^, ^2022^).

The purpose of the current study was to evaluate the marginal gap and fracture resistance of pulpally treated primary molars restored with 3D printed resin crowns in comparison to prefabricated zirconia crowns.

In vitro research was performed to create a controlled environment and get over certain restrictions related to clinical testing, such as individual human variation. Natural extracted teeth were used, as they mimic the actual force distribution at the inner surface of the crowns (Makawi and Khattab 2019).

The specimens were subjected to thermomechanical aging to simulate approximately 6 months of clinical performance. Additionally, the measurements of marginal gaps and fracture resistance were decided to be done after the cementation of crowns to simulate the clinical conditions (Al-Shibri and Elguindy 2017).

Marginal adaptation is essential for clinical acceptance of full coverage crowns. Inadequate crown marginal adaptation may lead to gingivitis, hypersensitivity, and recurrent caries by increasing the quantity of retained plaque (Al-Haj 2019). Holmes et al. (1989) defined the external marginal adaptation as the vertical marginal misfit measured parallel to the path of insertion of the crown, which can be made from points along the external surface of the crown to the axial wall of the preparation. Regarding crown longevity, most authors agree that a marginal gap between 100 and 150 μm is the range of clinical acceptance (Al-Haj 2019; Çakmak et al. 2021).

The marginal gap was evaluated using a stereomicroscope at × 40 magnification. Eight measurements were made for each axial surface of each crown, and this number was enough to give a consistent estimate of the gap size (Rastogi and Kamble 2011).

The results revealed that prefabricated zirconia crowns had a higher mean value of marginal gap (34.14 ± 9.79 µm) than 3D printed microfilled hybrid crowns (32.00 ± 7.54 µm).

The results of the current study were in line with the results of an in vitro study conducted by Kumar et al. (2021), who evaluated the marginal fit of two interim restorations fabricated by 3D printing and milling techniques and showed that 3D printed restorations exhibit significantly lower marginal and internal gap values than CAD/CAM milled counterparts.

The current study’s findings were consistent with those of the study by Peng et al. (2020), who measured the marginal discrepancy of resin crowns fabricated manually, as well as using CAD/CAM and 3D printing techniques. They reported that the resin crowns fabricated by 3D printing and CAD/CAM had smaller marginal discrepancy than the manually fabricated crowns.

Also, this study's results agree with a study conducted by Salman et al. (2021), who used a 20 × stereomicroscope to investigate the external marginal gap of prefabricated zirconia crowns (Nusmile) following cementation with self-adhesive resin cement. The results showed that there was a high external marginal gap with zirconia crowns in comparison to preformed metal crown and suggested searching for alternatives.

Conversely, Al-Haj (2019) assessed and compared the marginal and internal fit of PMCs with those of pre-veneered preformed metal crowns and prefabricated zirconia crowns using different luting cements. The mean marginal gap width for zirconia crowns was 0.17 ± 0.09, which was much less than the values observed in the current study. This difference in results might be due to the different techniques utilized to measure the marginal gap. The authors in the previous study sectioned the crowns in a buccolingual direction, unlike the current study where the samples were examined from all aspects without sectioning.

Regarding the results of fracture resistance, it was found that 3D printed microfilled hybrid resin crowns had a significantly higher value (1235.97 ± 412.12 N) than prefabricated zirconia crowns (576.56 ± 221.53 N). These values are significantly higher than the physiologic maximum occlusal force (500 N), which varies depending on the patient's age and facial morphology (Al-Shibri and Elguindy 2017).

Owais et al. (2013) reported that the maximum occlusal biting force for the different dentition stages are about 176 N in the early primary stage, 240 N in the late primary stage, 289 N in the early mixed stage, 433 N in the late mixed stage, and 527 N in the permanent dentition stage. Therefore, it can be concluded that all the materials tested in this study could withstand the maximum intraoral occlusal bite forces of mastication in primary and mixed dentition.

The current study's findings are consistent with those of Corbani et al. (2020), who investigated the fracture resistance of 3D printed resin crowns and reported that 3D printed crowns with 1.0 mm thickness showed high values (1945.9 ± 65.32 N) compared to the milled crowns.

Also, this study's results are in agreement with those of other studies conducted by Al-Halabi et al. (2020) and Kim et al. (2022), who reported that the force values required to fracture 3D printed resin crowns were 1495.05 ± 320.675 N and 1634.4 ± 289.3 N, respectively.

Conversely, Abbas et al. (2024) conducted an in vitro study to compare the marginal gap and fracture resistance of 3D printed endocrowns and prefabricated zirconia crowns for the restoration of pulpotomized primary molars and reported that the marginal gap of prefabricated zirconia crowns had significantly higher values than endocrowns and no significant difference between both groups regarding fracture resistance (*P* = 0.527). This difference in the results might be due to different designs of crowns between this study and the current study.

Based on the methods and results of this study, it is possible to provide an additional esthetic solution for parents/children to satisfy the need for esthetic restoration of primary molars.

Higher fracture resistance values indicate that 3D printed crowns can withstand the occlusal forces in children. A high resistance to fractures can reduce the incidence of crown failure, contributing to better clinical outcomes and less frequent follow-up appointments for replacements. Furthermore, improved marginal adaptation of 3D printed crowns may contribute to improved long-term outcomes by minimizing the potential for bacterial infiltration and ensuring better crown retention.

However, the current study still has some limitations. The in vitro design standardizes all the variables that are not possible in clinical practice. Another limitation is that the sample size could have been larger to increase the accuracy of the results and the validity of the study. Additionally, the final thickness of the 3D printed crowns and the prefabricated crowns was not standardized, which is a critical factor to consider that can potentially affect gingival health. Also sectioning of the teeth would have provided better visibility in marginal gap assessment.

Further research with a larger sample size should be conducted on other mechanical properties of the 3D printed crowns, such as wear resistance, fatigue strength, permeability, solubility, staining over time, and whether physical properties or adhesion strength changes over time. A more comprehensive assessment of full coverage crowns may be achieved through the inclusion of recently launched preformed resin crowns as an independent comparative group in future studies. An in vivo study should also be conducted to verify the findings clinically.

## Conclusion

Considering the limitations of the present in vitro study, it has been shown that there were significant differences in fracture resistance among the experimental groups, as 3D printed microfilled hybrid crowns had significantly higher mean values than prefabricated zirconia crowns. However, the mean fracture resistance values of both crowns exceeded the maximum biting forces of children and adolescents, indicating their efficient clinical applicability.

Regarding the marginal gap, there was no significant difference between the experimental groups, suggesting that 3D printed microfilled hybrid resin crowns could be an acceptable alternative to prefabricated zirconia crowns.

## Data Availability

The data that support the findings of this study are available from the corresponding author upon request.

## Abbreviations

PMC: Preformed metal crown
ZC: Zirconia crown
3D printing: Three-dimensional printing technology
DLP: Digital light processing
CAD–CAM: Computer-aided design, and computer-aided manufacturing
µm: Micrometer

## References

[CR1] Abbas LH, Wassel MO, Hassan IT, El-Dimeery AG, ElGhazawy RK. In-vitro comparison of marginal gap and fracture resistance of prefabricated zirconia crowns and three dimensional- printed endocrowns for restoration of pulpotomized primary molars. Int J Chem Biochem Sci. 2024;25(14):89–99.

[CR2] Agrawal R, Khanduja R, Singhal M, Gupta S, Kaushik M. Clinical evaluation of stainless steel crown versus zirconia crown in primary molars: an in vivo study. Int J Clin Pediatr Dent. 2022;15(1):15–9. 10.5005/jp-journals-10005-2134. 35528499 10.5005/jp-journals-10005-2134 PMC9016922

[CR3] Ahmad SM, Dawood SN, Dalloo GAM, Al-Barazanchi TRH. Evaluation of mechanical properties of different polyetheretherketone endodontic post systems: an in vitro study. BMC Oral Health. 2023;23(1):537. 10.1186/s12903-023-03193-7. 37542242 10.1186/s12903-023-03193-7 PMC10401823

[CR4] Ajayakumar LP, Chowdhary N, Reddy VR, Chowdhary R. Use of restorative full crowns made with zirconia in children: a systematic review. Int J Clin Pediatr Dent. 2020;13(5):551–8. 10.5005/jp-journals-10005-1822. 33623346 10.5005/jp-journals-10005-1822 PMC7887175

[CR5] Al-Haj AS. In vitro comparison of marginal and internal fit between stainless steel crowns and esthetic crowns of primary molars using different luting cements. Dent Res J (Isfahan). 2019;16(6):366–71. 31803381 PMC6873236

[CR6] Al-Halabi MN, Bshara N, Comisi JC, Nassar JA. Evaluation of fracture resistance force in three types of primary molar crowns: milled by CAD\CAM, 3D dental printed, and composite celluloid crowns. Eur Dent Res Biomater J. 2020;1:33–9. 10.1055/s-0040-1716944.

[CR7] Al-Halabi MN, Bshara N, Nassar JA, Comisi JC, Alawa L. Comparative assessment of novel 3D printed resin crowns versus direct celluloid crowns in restoring pulp treated primary molars. J Evid-Based Dent Pract. 2022;22(1):101664. 10.1016/j.jebdp.2021.101664. 35219462 10.1016/j.jebdp.2021.101664

[CR8] Alshamrani A, Alhotan A, Owais A, Ellakwa A. The clinical potential of 3D-printed crowns reinforced with zirconia and glass silica microfillers. J Funct Biomater. 2023;14(5):267. 10.3390/jfb14050267. 37233377 10.3390/jfb14050267 PMC10218850

[CR9] Al-Shibri S, Elguindy J. Fracture resistance of endodontically treated teeth restored with lithium disilicate crowns retained with fiber posts compared to lithium disilicate and cerasmart endocrowns: in Vitro study. Dentistry. 2017;7(12):464.

[CR10] Alzanbaqi SD, Alogaiel RM, Alasmari MA, et al. Zirconia crowns for primary teeth: a systematic review and meta-analyses. Int J Environ Res Public Health. 2022;19(5):2838. 10.3390/ijerph19052838. 35270531 10.3390/ijerph19052838 PMC8910015

[CR11] Bani-Hani T, Al-Fodeh RS, Al-Wahadni AM, Abu-Alhaija ES, Al-Hakam M. An in-vitro investigation into the fracture resistance of prefabricated and custom-made zirconia crowns for permanent molars in children. Dent J. 2025;13(2):64. 10.3390/dj13020064. 10.3390/dj13020064 PMC11854477 39996938

[CR12] Beattie S, Taskonak B, Jones J, Chin J, Sanders B, Tomlin A, Weddell J. Fracture resistance of 3 types of primary esthetic stainless steel crowns. Canad Dental Assoc J. 2011;7:90. 21736864

[CR13] Çakmak G, Cuellar AR, Donmez MB, Schimmel M, Abou-Ayash S, Lu W-E, Yilmaz B. Effect of printing layer thickness on the trueness and margin quality of 3D-printed interim dental crowns. Appl Sci. 2021;11(19):9246. 10.3390/app11199246.

[CR14] Clark L, Wells MH, Harris EF, Lou J. Comparison of amount of primary tooth reduction required for anterior and posterior zirconia and stainless steel crowns. Pediatr Dent. 2016;38(1):42–6. 26892214

[CR15] Corbani K, Hardan L, Skienhe H, Özcan M, Alharbi N, Salameh Z. Effect of material thickness on the fracture resistance and failure pattern of 3D-printed composite crowns. Int J Comput Dent. 2020;23(3):225–33. 32789310

[CR16] El Makawi Y, Khattab N. In vitro comparative analysis of fracture resistance of lithium disilicate endocrown and prefabricated zirconium crown in pulpotomized primary molars. Open Access Maced J Med Sci. 2019;7(23):4094–100. 32165959 10.3889/oamjms.2019.864 PMC7061377

[CR17] Faul F, Erdfelder E, Lang AG, Buchner A. G*Power 3: a flexible statistical power analysis program for the social, behavioral, and biomedical sciences. Behav Res Methods. 2007;39(2):175–91. 10.3758/bf03193146. 17695343 10.3758/bf03193146

[CR18] Goswami M, Jangra B, Chauhan N, Khokhar A. Esthetics in pediatric dentistry-BioFlx crowns: case series. Int J Clin Pediatr Dent. 2024;17(3):357–61. 39144501 10.5005/jp-journals-10005-2766 PMC11320795

[CR19] Hamrah MH, Mokhtari S, Hosseini Z, Khosrozadeh M, Hosseini S, Ghafary ES, Hamrah MH, Tavana N. Evaluation of the clinical, child, and parental satisfaction with zirconia crowns in maxillary primary incisors: a systematic review. Int J Dent. 2021. 10.1155/2021/7877728. 34285695 10.1155/2021/7877728 PMC8275371

[CR20] Hanafi L, Altinawi M, Comisi JC. Evaluation and comparison two types of prefabricated zirconia crowns in mixed and primary dentition: a randomized clinical trial. Heliyon. 2021;7(2): e06240. 10.1016/j.heliyon.2021.e06240. 33665422 10.1016/j.heliyon.2021.e06240 PMC7900688

[CR21] Holmes JR, Bayne SC, Holland GA, Sulik WD. Considerations in measurement of marginal fit. J Prosthet Dent. 1989;62(4):405–8. 2685240 10.1016/0022-3913(89)90170-4

[CR22] Jang G, Kim SK, Heo SJ, Koak JY. Fit analysis of stereolithography-manufactured three-unit resin prosthesis with different 3D-printing build orientations and layer thicknesses. J Prosthet Dent. 2024;131(2):301–12. 10.1016/j.prosdent.2021.11.031. 36653209 10.1016/j.prosdent.2021.11.031

[CR23] Kaur K, Suneja B, Jodhka S, Saini RS, Chaturvedi S, Bavabeedu SS, Alhamoudi FH, Cicciù M, Minervini G. Comparison between restorative materials for pulpotomised deciduous molars: a randomized clinical study. Children. 2023;10(2):284. 10.3390/children10020284. 36832414 10.3390/children10020284 PMC9955046

[CR24] Kim N, Kim H, Kim IH, Lee J, Lee KE, Lee HS, Kim JH, Song JS, Shin Y. Novel 3D printed resin crowns for primary molars: in vitro study of fracture resistance, biaxial flexural strength, and dynamic mechanical analysis. Children (Basel Switzerland). 2022;9(10):1445. 10.3390/children9101445. 36291379 10.3390/children9101445 PMC9600781

[CR25] Kumar R, Suganna M, Ahmed R, et al. An in vitro evaluation of 3d-printed provisional restoration marginal adaptation on diverse finish lines. Int J Prosthod Restor Dent. 2021;11:82–7. 10.5005/jp-journals-10019-1313.

[CR26] Lee KE, Hyun SK, Seo YS, Taeyang L, Hyo-Seol L, Je SS. Comparison of three-dimensional printed resin crowns and preformed stainless steel crowns for primary molar restorations: a randomized controlled trial. J Clin Pediatr Dent. 2024;48(3):59–67. 38755983 10.22514/jocpd.2024.060

[CR27] Mahfouz Omer SM, El-Sherbiny RH, El-Desouky SS. Effect of N-acetylcysteine on initial carious enamel lesions in primary teeth: an in-vitro study. BMC Oral Health. 2023;23(1):520. 10.1186/s12903-023-03224-3. 37491222 10.1186/s12903-023-03224-3 PMC10369821

[CR28] Mathew MG, Roopa KB, Soni AJ, Khan MM, Kauser A. Evaluation of clinical success, parental and child satisfaction of stainless steel crowns and zirconia crowns in primary molars. J Fam Med Prim Care. 2020;9(3):1418–23. 10.4103/jfmpc.jfmpc_1006_19. 10.4103/jfmpc.jfmpc_1006_19 PMC7266243 32509626

[CR29] Owais AI, Shaweesh M, Abu Alhaija ESJ. Maximum occusal bite force for children in different dentition stages. Eur J Orthod. 2013;35(4):427–33. 10.1093/ejo/cjs021. 22518063 10.1093/ejo/cjs021

[CR30] Park S, Cho W, Lee H, et al. Strength and surface characteristics of 3D-printed resin crowns for the primary molars. Polymers (Basel). 2023;15(21):4241. 10.3390/polym15214241. 37959921 10.3390/polym15214241 PMC10648608

[CR31] Peng CC, Chung KH, Ramos V Jr. Assessment of the adaptation of interim crowns using different measurement techniques. J Prosthodont. 2020;29(1):87–93. 10.1111/jopr.13122. 31702087 10.1111/jopr.13122

[CR32] Rastogi A, Kamble V. Comparative analysis of the clinical techniques used in evaluation of marginal accuracy of cast restoration using stereomicroscopy as gold standard. J Adv Prosthodont. 2011;3(2):69–75. 10.4047/jap.2011.3.2.69. 21814614 10.4047/jap.2011.3.2.69 PMC3141121

[CR33] Refaie A, Fouda A, Bourauel C, Singer L. Marginal gap and internal fit of 3D printed versus milled monolithic zirconia crowns. BMC Oral Health. 2023;23(1):448. 10.1186/s12903-023-03184-8. 37403169 10.1186/s12903-023-03184-8 PMC10318718

[CR34] Salman NR, Khattab NMA, Gomaa YFEA. Assessment of the external marginal adaptation of prefabricated zirconia crowns for restoring primary molars using a stereomicroscope. Ann Roman Soc Cell Biol. 2021;25(6):20218–26.

[CR35] Schweiger J, Edelhoff D, Güth JF. 3D printing in digital prosthetic dentistry: an overview of recent developments in additive manufacturing. J Clin Med. 2021;10(9):21010. 10.3390/jcm10092010. 10.3390/jcm10092010 PMC8125828 34067212

[CR36] Simsek H, Derelioglu S. In vitro comparative analysis of fracture resistance in inlay restoration prepared with CAD-CAM and different systems in the primary teeth. BioMed Res Int. 2016. 10.1155/2016/4292761. 27830145 10.1155/2016/4292761 PMC5086510

[CR37] Team RC. R Core Team 2023 R: a language and environment for statistical computing. R foundation for statistical computing. 2023. https://www.R-project.org/.

